# *Mycobacterium abscessus* biofilms have viscoelastic properties which may contribute to their recalcitrance in chronic pulmonary infections

**DOI:** 10.1038/s41598-021-84525-x

**Published:** 2021-03-03

**Authors:** Erin S. Gloag, Daniel J. Wozniak, Paul Stoodley, Luanne Hall-Stoodley

**Affiliations:** 1 Department of Microbial Infection and Immunity, The Ohio State University, 711 Biomedical Research Tower, 460 W 12th Avenue, Columbus, OH USA; 2 Department of Microbiology, The Ohio State University, Columbus, OH 43210 USA; 3 Department of Orthopedics, The Ohio State University, Columbus, OH 43210 USA; 4 National Biofilm Innovation Centre (NBIC) and National Centre for Advanced Tribology at Southampton (nCATS), University of Southampton, Southampton, SO17 1BJ UK

**Keywords:** Biophysics, Microbiology

## Abstract

*Mycobacterium abscessus* is emerging as a cause of recalcitrant chronic pulmonary infections, particularly in people with cystic fibrosis (CF). Biofilm formation has been implicated in the pathology of this organism, however the role of biofilm formation in infection is unclear. Two colony-variants of *M. abscessus* are routinely isolated from CF samples, smooth (*Ma*^Sm^) and rough (*Ma*^Rg^). These two variants display distinct colony morphologies due to the presence (*Ma*^Sm^) or absence (*Ma*^Rg^) of cell wall glycopeptidolipids (GPLs). We hypothesized that *Ma*^Sm^ and *Ma*^Rg^ variant biofilms might have different mechanical properties. To test this hypothesis, we performed uniaxial mechanical indentation, and shear rheometry on *Ma*^Sm^ and *Ma*^Rg^ colony-biofilms. We identified that *Ma*^Rg^ biofilms were significantly stiffer than *Ma*^Sm^ under a normal force, while *Ma*^Sm^ biofilms were more pliant compared to *Ma*^Rg^, under both normal and shear forces. Furthermore, using theoretical indices of mucociliary and cough clearance, we identified that *M. abscessus* biofilms may be more resistant to mechanical forms of clearance from the lung, compared to another common pulmonary pathogen, *Pseudomonas aeruginosa.* Thus, the mechanical properties of *M. abscessus* biofilms may contribute to the persistent nature of pulmonary infections caused by this organism.

## Introduction

In the cystic fibrosis (CF) lung, due to genetic mutations in the cystic fibrosis transmembrane regulator (CFTR) ion channel responsible for this disease, the mucus lining of the airways becomes dehydrated and highly viscous, leading to impaired clearance and accumulation. This provides a niche that can be readily colonized by inhaled microorganisms, which form biofilm aggregates within the accumulated mucus layer^1,2^. These biofilms lead to recurrent progressive infections, inflammation, bronchiectasis and, eventually, respiratory failure. Nontuberculous mycobacteria (NTM) are an increasingly common complication in CF, now ranking as the third most frequent cause of lung infection in people with CF^3–5^. *Mycobacterium abscessus* infection is particularly challenging, infecting younger people with CF and resulting in a poorer prognosis than those infected with other NTM^3,4,6–8^. However, the mechanisms underlying the rising incidence of *M. abscessus* infection in people with CF remain ill-defined^9,10^.

*Mycobacterium abscessus* has two distinct colony variants, based on the presence (smooth morphotype; *Ma*^Sm^) or absence (rough morphotype; *Ma*^Rg^) of cell wall glycopeptidolipids (GPLs)^11,12^. The prevailing view of chronic *M. abscessus* infection is that *Ma*^Sm^ is a noninvasive, biofilm-forming, persistent phenotype, and *Ma*^Rg^ is an invasive phenotype that is unable to form biofilms. We have previously shown, however, that *Ma*^Rg^ is hyper-aggregative and is capable of forming biofilm aggregates, which are significantly more tolerant than planktonic *M. abscessus* to acidic pH, hydrogen peroxide, or antibiotic treatment^11^. These studies indicate that development of biofilm aggregates contribute to the persistence of *M. abscessus* in the face of antimicrobial agents, regardless of morphotype. Antibiotic regimens that reliably cure *M. abscessus* infections are lacking. A better understanding of the the biofilm biology of this organism and its ability to cause persistent pulmonary infections is needed to explore new treatment strategies^13,14^.

The study of biofilm mechanics has gained interest, as it is being realized that these properties are important for understanding biofilm biology and how biofilms respond to chemical and mechanical forms of eradication^15^. Interestingly, changes in colony morphology have been correlated to differences in biofilm formation^16^, and differences in biofilm mechanics^17,18^. We therefore hypothesized that the biofilms of *Ma*^Sm^ and *Ma*^Rg^ may have different mechanical properties, due to their distinct colony morphologies, and that these properties may help to account for the persistence of infection caused by this organism. In the present study, we used mechanical indentation and shear rheometry to evaluate the viscoelastic properties of *Ma*^Sm^ and *Ma*^Rg^ biofilms. We also use known concepts of mucus viscoelasticity to predict how effectively *M. abscessus* biofilms may be cleared from the lung by mucociliary and cough clearance. Our findings support the hypothesis that biofilm viscoelasticity could contribute to the persistence of biofilm-associated infefctions^15,19^, and provides novel insight into why *M. abscessus* may be such a persistent pathogen in airway infection in people with CF.

## Results

### *Ma *colony-biofilms maintain the distinct morphologies of *Ma*^Sm^ and *Ma*^Rg^ variants

**Figure 1:**
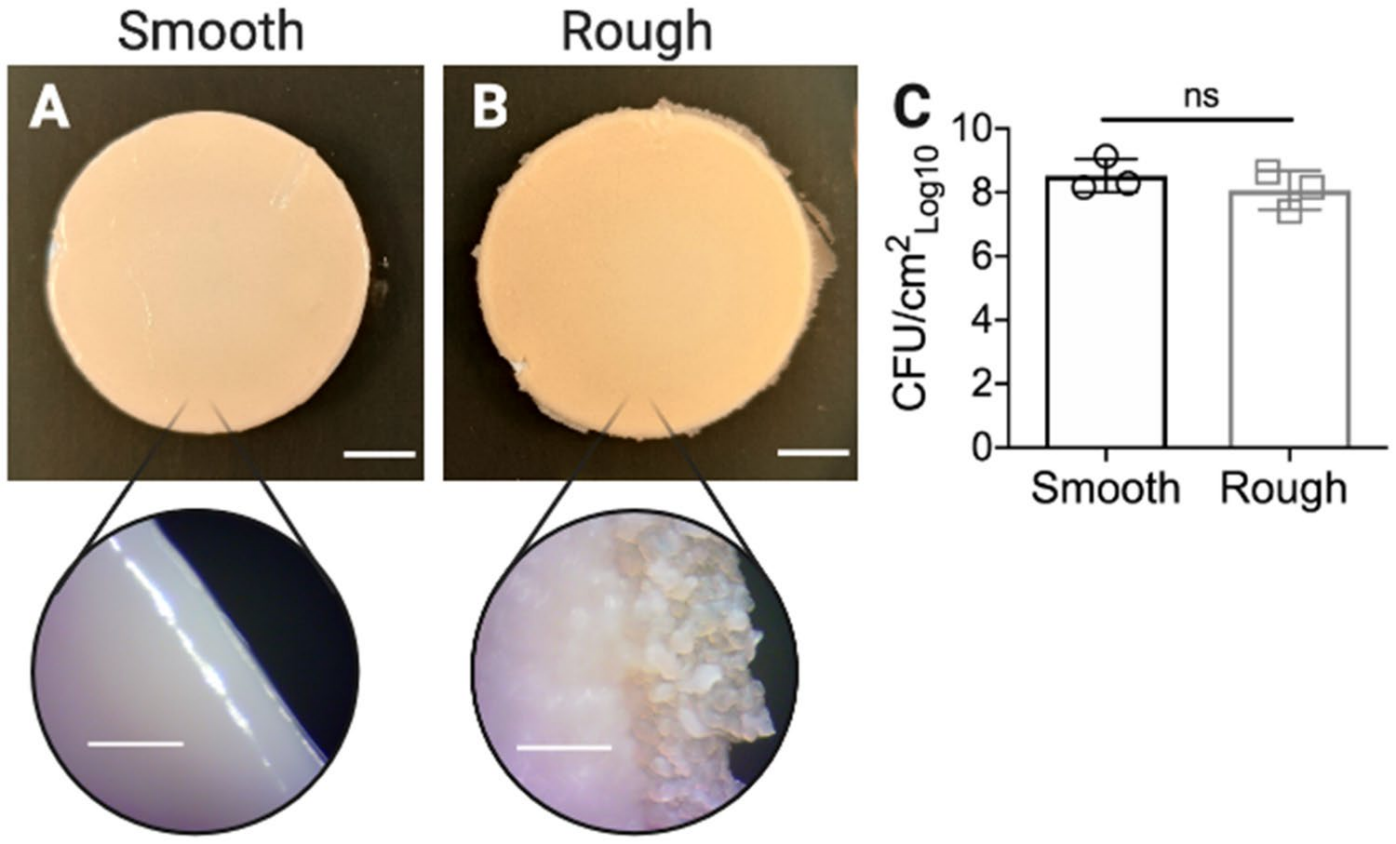
Morphology of *Ma*^*Sm*^ and *Ma*^*Rg*^ colony-biofilms. **(A)** *Ma*^*Sm*^ and **(B)** *Ma*^*Rg*^ were grown on nitrocellulose membranes for 4 days, transferring the membranes onto fresh media after 48 h. Colony-biofilms were then imaged to visualize the macroscopic morphology. Scale bar indicates 5 mm, and 0.5 mm for the zoomed insets. **(C)** To assess the biomass and if each variant was stable during biofilm formation, 4 day M*. abscessus* biofilms were enumerated for CFUs. Evidence of phenotypic changes of the colony morphologies for either was not observed at 37ºC over 4 days. Statistical analysis was performed using a Student’s t-test; ns indicated not significant. N = 3.

*Mycobacterium abscessus* is a rapidly growing NTM, typically showing colony morphology by 4 days on agar media. To test the hypothesis that biofilms of *Ma*^Sm^ and *Ma*^Rg^ variants might differ in their mechanical properties, uniaxial indentation and shear rheology was performed on 4 day M*. abscessus* colony-biofilms (See Supplementry Methods). As this is the first time that we have used this biofilm model for *M. abscessus,* we examined the colony-biofilms after 4 days of growth (Fig. 1). Macroscopically, biofilm formation was evident, covering the filter. Biofilm morphology maintained the characteristic phenotypes of each variant^11^. That is, biofilms of *Ma*^Sm^, had a smooth, almost mucoid appearance (Fig. 1A), while *Ma*^*Rg*^ biofilms showed a cauliflower-like morphology (Fig. 1B) in agreement with our previous study showing cording at the periphery of *Ma*^*Rg*^ colonies^11^. Importantly, when these colony-biofilms were disrupted, and plated to observe single cells, the number of cells within the biofilm was similar across the two variants, and no reversion across phenotypes was observed during this time (Fig. 1C), demonstrating that during the 4 day biofilm growth period, each variant was stable.

### *Ma*^Rg^ biofilms are stiffer than *Ma*^Sm^ biofilms in uniaxial compression

**Figure 2:**
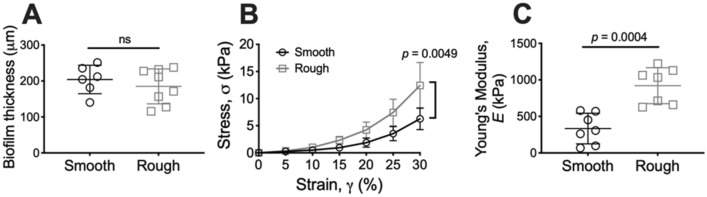
*Ma*^Rg^ colony-biofilms are stiffer compared to *Ma*^Sm^ biofilms in compression. **(A)** Thickness and **(B)** stress–strain curves of 4 day M*. abscessus* colony-biofilms determined from uniaxial indentation analysis. **(C)** Young’s modulus of *M. abscessus* biofilms, determined from the lower linear potion of the force–displacement curve, corresponding to 0–30% strain. The full stress–strain curve, up to 100% strain is represented in Fig. S1. N = 4; data presented as individual data points with mean ± SD. Statistical analysis was performed using a Student’s t-test; ns indicates not significant.

To determine the stiffness of *M. abscessus* biofilms under a normal force (force that is applied perpendicular to the surface), uniaxial indentation was performed on 4 day M*. abscessus* colony-biofilms. During this analysis, biofilms are compressed and the required force is measured. This analysis is also used to determine the biofilm thickness, which revealed that biofilms of each variant were of similar thickness (Fig. 2A), consistent with the observation that biofilms had a similar number of CFUs (Fig. 1C). Stress–strain curves revealed that *Ma*^Sm^ and *Ma*^Rg^ biofilms displayed a ‘J-shaped’ curve, where the biofilms became progressively stiffer as they were compressed (Fig. S1). Stress–strain curves also revealed that there were significant mechanical differences between *Ma*^Sm^ and *Ma*^Rg^ biofilms (Fig. 2B; *p* = 0.0049). To quantify these differences, the Young’s modulus was determined from the lower linear portions of the curve, which revealed that the Young’s modulus of *Ma*^Rg^ biofilms was approximately two-fold greater than *Ma*^Sm^ biofilms. This indicates that *Ma*^Rg^ biofilms were significantly stiffer under uniaxial compression, compared to *Ma*^Sm^ biofilms (Fig. 2C; *p* = 0.0004).

### *M. abscessus* biofilms are highly viscoelastic and *Ma*^Sm^ biofilms are more pliant than *Ma*^Rg^ under shear

**Figure 3:**
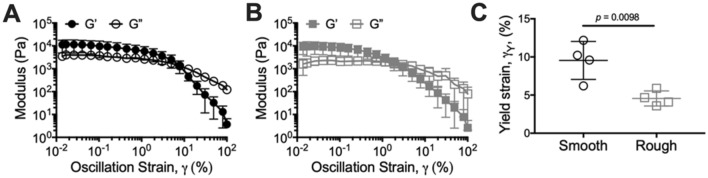
*Ma*^Sm^ colony-biofilms are more pliant than *Ma*^Rg^ biofilms in shear. Strain sweep profiles of 4 day **(A)** *Ma*^Sm^ and **(B)** *Ma*^Rg^ colony-biofilms. N = 4; data presented as mean ± SD; G’ = storage modulus and G” = loss modulus. **(C)** Yield strain of *M. abscessus* colony-biofilms determined from **(A)** and **(B)**. N = 4; data presented as individual data points with mean ± SD. Statistical analysis was performed using a Student’s t-test.

To examine the mechanical properties of *M. abscessus* colony-biofilms in further detail, *Ma*^Sm^ and *Ma*^Rg^ biofilms were analyzed using spinning-disc rheology. For these analyses a shear force (a force that is applied parallel to a surface) is applied and the resulting stress or strain is measured (See Supplementary Methods). Oscillatory strain sweeps were performed, where the oscillatory strain was incrementally increased and the storage (G’) and loss (G”) moduli measured, which reflect the elastic and viscous response, respectively^15^. The measured storage and loss moduli plateaus were similar for *Ma*^Sm^ and *Ma*^Rg^ biofilms (Fig. 3A,B), suggesting that biofilms of each variant behaved similarly within the linear viscoelastic region. From this analysis we determined the yield strain, which represents the strain where the biofilm integrity begins to break down, due to the increasing applied strain, and transitions to fluid-like behavior^20^. Interestingly, the yield strain of *Ma*^Sm^ biofilms was significantly greater than *Ma*^Rg^ biofilms (Fig. 3C; *p* = 0.0098). This suggests that *Ma*^Sm^ biofilms are more pliant than *Ma*^Rg^ biofilms, and that *Ma*^Sm^ biofilms can be deformed to a greater extent before cohesive failure occurs. This is also consistent with the Young’s modulus of these biofilms, which indicated that *Ma*^Sm^ biofilms can be compressed more readily compared to *Ma*^Rg^ biofilms (Fig. 2C).

**Figure 4:**
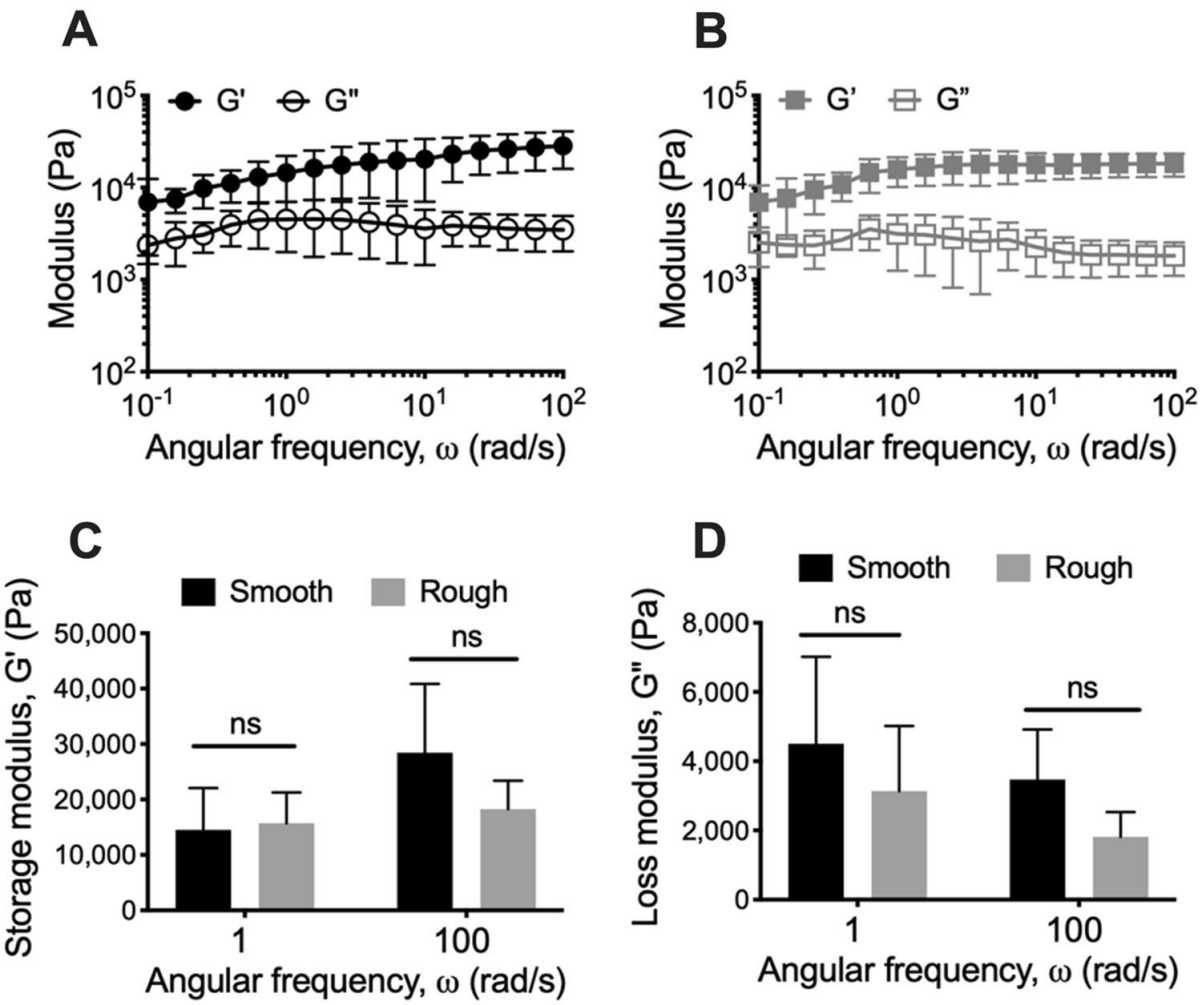
*M. abscessus* colony-biofilms are highly viscoelastic. Frequency sweep profiles of 4 day **(A)** *Ma*^Sm^ and **(B)** *Ma*^Rg^ colony-biofilms. G‘ = storage modulus and G” = loss modulus **(C)** Storage and **(D)** and loss moduli values at 1 and 100 rad/s. N = 4; data presented as mean ± SD. Statistical analysis was performed using a Student’s t-test; ns indicates not signficant.

Oscillatory frequency sweeps were also performed, where the oscillatory frequency was incremented under a consistent applied strain, and the storage and loss moduli measured. Across the range of frequencies analyzed, the storage and loss moduli were relatively independent of frequency, with the storage modulus greater than the loss modulus for both *M. abscessus* variant biofilms (Fig. 4A,B). This indicates that both *Ma*^Sm^ and *Ma*^Rg^ biofilms displayed elastic behavior that was dominant across the analyzed conditions. There were no significant differences between the mechanical behavior of *Ma*^Sm^ and *Ma*^Rg^ biofilms using this analysis (Fig. 4C,D). However, compared to *Ma*^Sm^ biofilms, *Ma*^Rg^ biofilms trended toward a reduced loss modulus across the analyzed frequencies (Fig. 4D).

### Theoretical indices suggest that *M. abscessus* biofilms, independent of morphotype, may resist clearance from the lung

**Figure 5:**
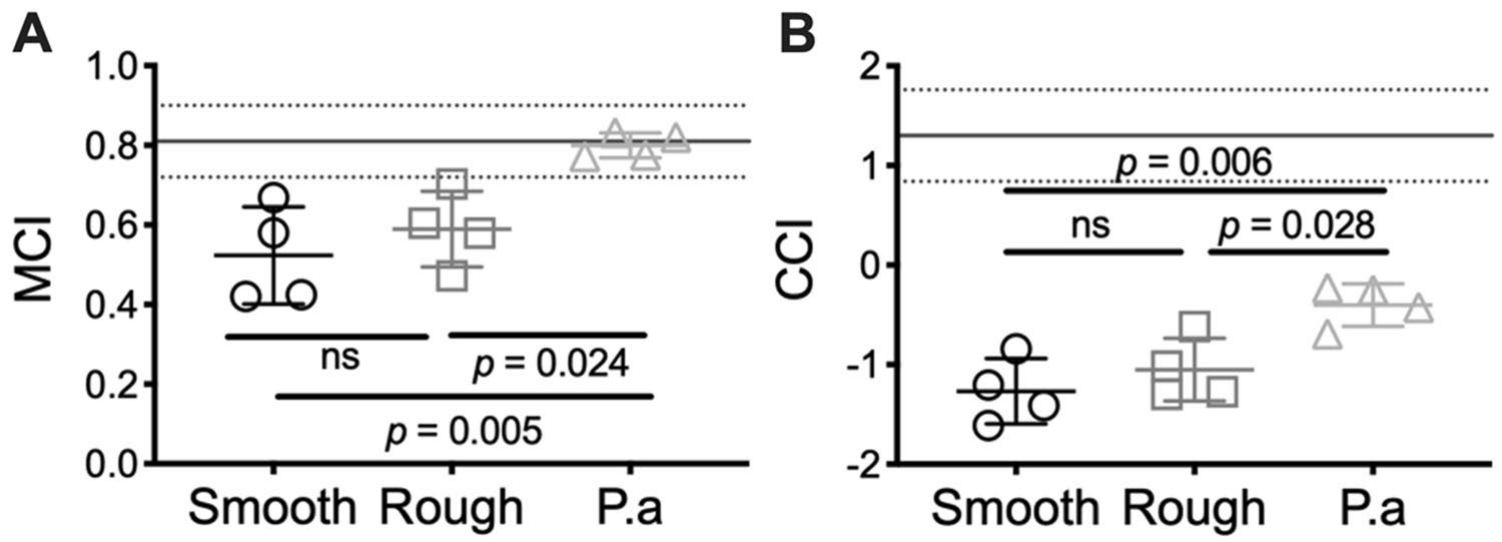
*M. abscessus* colony-biofilms have reduced theoretical mucociliary and cough clearance index. **(A)** Mucociliary and **(B)** cough clearance index of 4-day *M. abscessus* and *P. aeruginosa* (P.a) colony-biofilms. Lines at **(A)** and **(B)** are the MCI (0.81 ± 0.09) and CCI (1.3 ± 0.46), respectively, of sputum collected from patients with CF, previously determined by^54^. N = 4; data presented as individual data points with mean ± SD. Statistical analysis was performed using a one-way ANOVA with a Tukey’s post-hoc test; ns indicates not significant.

To determine if the mechanical properties of *M. abscessus* biofilms may correlate with recalcitrance of *M. abscessus* in pulmonary infections, we calculated the mucociliary (MCI) and cough (CCI) clearance index, according to Eqs. (2) and (3), using values from the frequency sweep analysis of these biofilms (Fig. 4). The MCI and CCI were developed to correlate sputum viscoelasticity to predicted levels of clearance from the lung via either mechanism^21,22^. The MCI and CCI of *Ma*^Sm^ and *Ma*^Rg^ biofilms were similar. However, due to the highly viscoelastic behavior of both colony variant biofilms (Fig. 4A,B), the indices were less than the MCI and CCI reported for sputum collected from people with CF (Fig. 5; line).

To compare *M. abscessus* biofilms to the biofilms of another common pathogen causing chronic pulmonary infections in people with CF, we analyzed 4 day wild type *Pseudomonas aeruginosa* colony-biofilms^23^. *P. aeruginosa* is considered a model organism for biofilm formation, and we have previously determined the MCI and CCI for this organism^17^. Using the same testing conditions for *M. abscessus* biofilms described here, we performed oscillatory frequency sweeps on 4 day wild type *P. aeruginosa* colony-biofilms (See Supplmentary Results; Fig. S2), and similarly determined the MCI and CCI for comparison (Fig. 5). The indices of *Ma*^Sm^ and *Ma*^Rg^ biofilms were significantly less than that determined for wild type *P. aeruginosa* biofilms (Fig. 5). This suggests that *M. abscessus* biofilms may be more resistant to mechanical clearance from the lung, compared to other common pulmonary pathogens, such as *P. aeruginosa*.

## Discussion

Compared to other biofilm forming organisms, such as *P. aeruginosa*, the understanding of biofilm formation and the role of biofilms in infection of NTMs is limited. It is important to understand biofilm mechanics from a biofilm biology stand-point, as these properties dictate the stability of the three-dimensional community structure^15^. We therefore set out to analyze the mechanical properties of *M. abscessus *in vitro biofilms to gain a better understanding of the biofilm biology of this organism. We found that *Ma*^Rg^ colony-biofilms were stiffer under normal forces (Fig. 2C), while *Ma*^Sm^ colony-biofilms were more pliable under both normal and shear forces (Figs. 2C, 3C). In constrast to the uniaxial indentation analysis (Fig. 2), oscillatory frequency sweeps indicated that there were no significant difference in the viscoelasticity of *Ma*^*Sm*^ and *Ma*^*Rg*^ biofilms under shear forces (Fig. 4). This indicates that *M. abscessus* biofilms were anisotropic, in that they have different mechanical properties when exposed to either a normal or shear force.

Earlier research by others suggested that only *Ma*^Sm^ variants formed biofilms and speculated that GPL expression enhanced sliding motility in CF mucus, making it better adapted to behave as a colonizing, biofilm-forming phenotype^24,25^. However, we have previously shown that the rough morphotype is hyper-aggregative^11^, indicating that each colony morphology variant is capable of forming biofilm aggregates. The finding here that *Ma*^Sm^ colony-biofilms were more pliable under both normal and shear forces (Figs. 2C, 3C) suggests that GPL likely affects the mechanical properties of *M. abscessus* biofilms. Further studies are needed to better understand the relationship of *M. abscessus* cell wall determinants, such as lipids, GPLs, and extracellular polyermic substance (EPS) with biofilm function and is the focus of future studies.

Uniaxial indentation, revealed that *Ma*^Sm^ and *Ma*^Rg^ biofilms each displayed a ‘J-shaped’ curve in response to increasing compression (Fig. 2B). This response is typical of biological materials, and has previously been observed for a number of different types of bacterial biofilms^17,26–28^. The Young’s modulus of *Ma*^Sm^ and *Ma*^Rg^ colony-biofilms, determined here, is similar to that reported for *Streptococcus mutans* hydrated biofilms^29^, *P. aeruginosa* colony-biofilms^17^, and surface adhered *Staphylococcus aureus, Staphylococcus epidermidis* and *Streptococcus salivarius* cells^30^.

Interestingly, *Ma*^Sm^ and *Ma*^Rg^ colony-biofilms have a storage modulus, which describes the elastic solid-like behavior^31^, approximately tenfold greater than that previously observed for other bacterial biofilms (Fig. S2A)^17,18,20,32–40^. This suggests that *M. abscessus* biofilms, regardless of the morphotype, are highly viscoelastic, and much stiffer than biofilms formed by other bacterial pathogens. It would, therefore, be of interest to compare the mechanical properties of other NTM biofilms, as well as other biofilms that have a lipid dominant EPS, to determine the contributions of lipid content to the EPS and to the biofilm mechanical properties. It is interesting to speculate that different host niches in the lung (or other infection sites) may favor stiffer or softer biofilms depending on different shear and chemical micro environments. However, this requires further clinical work.

Viscoelasticity is a property common to biofilms, suggesting that it is an adaptive trait of these mircobial communites^15^. It has therefore been predicted that biofilm viscoelasticity, not only is important from a biofilm biology stand-point, but also when considering microbial survival^41^. Understanding how biofilm mechanics impacts microbial survival in an infection is still unclear. However, there is a growing consensus that these properties are important for resisting both mechanical and chemical methods of eradication^15,19^. To gain further insight into how the mechanics of *M. abscessus* biofilms may influence the recalictrance of these infections, we used known, well-established concepts of mucus viscoelasticity to draw new hypotheses that may be relevant to *M. abscessus* biofilms in vivo, in the context of mechanical clearance from the lung. Changes in mucus viscoelasticity in CF are attributed to mucus hyper-secretion and changes to mucus composition, impeding mucus clearance by both mucociliary and cough clearance^42^. MCI and CCI are theoretical indices that were developed from in vitro lung clearance models to correlate viscoelastic properties of expectorated mucus with predicted levels of clearance from the lung^21,22^. These indices have been used to assess the efficiency of mucolytics for therapy regimes for people with CF^43^. MCI predicts that mucus elasticity correlates to improved mucociliary clearance by promoting efficient cilia beating^21,44^, while CCI predicts that mucus viscosity correlates to improved cough clearance by promoting mucus-airflow interactions^44,45^. We previously used these known relationships between mucus viscoelasticity and clearance, and applied these concepts to *P. aeruginosa* colony-biofilms, to determine if the viscoelastic properties of bacterial biofilms could likewise theoretically impact mechanical clearance from the lung^17^. We identified that elastic non-mucoid *P. aeruginosa* biofilms had a reduced CCI, while mucoid *P. aeruginosa* biofilms that had a low viscosity, had both a reduced MCI and CCI^17^. Here we determined the MCI and CCI of *M. abscessus* biofilms, and observed that they had an even lower or negative CCI compared to *P. aeruginosa*, attributed to the high viscoelasticity of these biofilms (Fig. 5B). This indicates that *M. abscessus* biofilms of each morphotype may resist clearance from the lung by cough. These findings provide novel insight into possible reasons why *M. abscessus* is a persistent and difficult to treat pulmonary pathogen.

A longitudinal rheological study of sputum from CF patients indicated that both viscosity and elasticity increase during pulmonary exacerbations, suggesting that CF sputum viscoelasticity is linked to disease state and airflow obstruction^46^. By applying known concepts of mucus rheology to biofilm mechanics and infection, our findings provide a novel way to understand *M. abscessus* biofilms and their recalcitrance. Moving forward, it will be important to develop in vitro and in vivo models of biofilm-mucus interactions to explore the hypotheses drawn from this work. More broadly, the impact of evaluating bacterial biofilm mechanical properties offers new avenues for the treatment of intractable pulmonary infections. Disruption of *M. abscessus* aggregates increased susceptibility to several antibiotics^13^. Moreover, liposomes, lipid nanoparticles and nanoparticle lipid carriers are being evaluated for pulmonary use^47^. This approach has been used on biofilms of *Burkholderia cepacia* complex, as well as *S. aureus* and *P. aeruginosa* biofilms in vitro^48,49^. These products not only have the potential to reduce exposure to high concentrations of antimicrobial agents following aerosol administration by reducing toxicity, but particularly relevant to NTM antibiotic therapy, might ameliorate the severe side effects often associated with antimycobacterial treatment. This strategy could further be used to target NTM, which have a high lipid content, to facilitate cohesive failure of biofilm aggregates in the lung in adddtion to delivering targeted antibiotics.

## Materials and methods

### *M. abscessus* culture and biofilm model

The colony-biofilm model was used here due to the established utility of this model for rheological analysis of biofilms^17,50^ (See Supplementary Methods). Colony-biofilms were grown as previously described with modifications^17^. Briefly, *M. abscessus* cultures were prepared by inoculating a 10 μL loop of isolated *Ma*^Sm^ or *Ma*^Rg^ from 7H10 agar plates into 7H9 without tween media and a single cell suspension prepared as described^11^. Cultures were normalized to an OD_600_ of 0.15–0.2 and 100 μL was pipetted onto sterile nitrocellulose filter membranes (25 mm, 0.45 μm pore size; Millipore) and incubated at 37 °C, 5% CO_2_ under humidified conditions for 4 days. This time point was selected as it provided sufficent biomass necessary for rheological analysis, while maintaining the characteristic morphology of *Ma*^Sm^ or *Ma*^Rg^ variants. Colony-biofilms of PAO1 *P. aeruginosa* were grown as previously described^17^.

Colony-biofilms were imaged using a Stereo Microscope (AmScope) fitted with a Microscope Digital Color CMOS camera (AmScope). Images were processed in FIJI^51^. Figure was complied in Biorender.com.

To assess the number of CFU/cm^2^ on sterile membranes after 4 days, colony-biofilms were scraped and transferred to 5 mL of 7H9 + tween 80 and vortexed with glass beads to disperse cells from the biofilm^11^. Cell suspensions were serially diluted and enumerated for CFUs in triplicate on 7H10 agar plates. To determine if either variant converted/reverted to another colony variant during biofilm growth, colony morphology was also assessed and verified by sub-culturing three colonies per replicate to ensure that the colony morphology was stable. CFUs were plated to 10^8^ dilution and colonies were streaked to isolation to validate colony variants.

### Rheometry apparatus

A Discovery Hybrid Rheometer-2 with a heat exchanger attached to the Peltier plate (TA Instruments) was used for all rheological measurements. For uniaxial indentation and spinning disc measurements (See Supplementary Methods), the rheometer was fitted with an 8 mm and 25 mm sand-blasted Smart Swap geometry, respectively. All measurements were performed at 37 °C, with the Peltier plate covered with a moist Kimwipe to prevent dehydration of the colony-biofilm. TRIOS v4 (TA instruments) software was used.

### Uniaxial indentation measurements

Uniaxial indentation measurements were performed under compression using an approach rate of 1 μm/s. Two colony-biofilms were analyzed per biological replicate (total of 4 biofilms analyzed), with two measurements per biofilm. The point of contact with the biofilm was determined to be where the force began to increase after the initial point of pull-on adhesion. Force–displacement curves were converted to stress–strain curves, for ease of comparison, as previously described^17^. The Young’s modulus was calculated according to Eq. (1)^52^:

1 $$E=\frac{slope\cdot (1-{v}^{2})}{2r}$$

where *v* is the assumed Poisson’s ratio of a biofilm (*v* = 0.5)^27^ and *r* is the radius of the geometry. The slope is of the force–displacement curve, and was taken at the lower linear region corresponding to 0–30% strain where R^2^ ≥ 0.9.

### Spinning-disc rheology

To normalize for differences in biofilm thickness across replicates, prior to analysis, colony-biofilms were compressed to a normal force of 0.01 N. For all analyses a total of 4 biofilms were analyzed, 2 per biological replicate.

Strain sweeps were performed by incrementing the oscillatory strain from 0.01 to 100% at a frequency of 1 Hz. The yield strain was taken where the storage (G’) and loss (G”) modulus intersected. Frequency sweeps were performed by incrementing the oscillating frequency from 0.1 to 100 rad/s at a constant strain of 0.1%. This strain was determined to be within the linear viscoelastic region of the strain sweeps for both *Ma*^Sm^ and *Ma*^Rg^ colony-biofilms.

Theoretical mucociliary clearance index (MCI) and cough clearance index (CCI), developed from in vitro models of mucus clearance^32,33^, were calculated according to Eqs. (2) and (3), respectively, using the relationship between the complex modulus (G*) and tanδ values determined from frequency sweep measurements^21,22,53^. The MCI and CCI was calculated from values determined at an angular frequency of 1 rad/s and 100 rad/s, respectively.

2 $$MCI=1.62-\left(0.22\times log{G}_{1}^{*}\right)-\left(0.77\times tan{\delta }_{1}\right)$$

3 $$CCI=3.44-\left(1.07\times log{G}_{100}^{*}\right)+\left(0.89\times tan{\delta }_{100}\right)$$

### Statistical analysis

Data are presented as mean ± SD. Statistical significance was determined using either an One-way ANOVA with a Tukey’s post-hoc test or a Student’s t-test. Analyses were performed using GraphPad Prism v.7 (Graphpad Software). Statistical significance was determined using a p-value < 0.05.

## Supplementary Information

Supplementary Information.

## Data Availability

All data generated or analysed during this study are included in this published article (and its Supplementary Information files).
