# Supplementary material for: Mycobacterium abscessus biofilms have viscoelastic properties which may contribute to their recalcitrance in chronic pulmonary infections
Source: Sci Rep. 2021 Mar 3;11:5020. doi: 10.1038/s41598-021-84525-x (PMC7930093; doi:10.1038/s41598-021-84525-x)
Supplement: Supplementary file 1 — Supplementary Information. [file 41598_2021_84525_MOESM1_ESM.docx]

***Mycobacterium abscessus* biofilms have viscoelastic properties which may contribute to their recalcitrance in chronic pulmonary infections.**

**Supplemental Information**

Erin S. Gloag^1^, Daniel J. Wozniak^1,2^, Paul Stoodley^1,3,4^, Luanne Hall-Stoodley^1^*

**Supplemental Methods**

**Rationale for using the colony-biofilm model**

Traditional biofilm models, such as flow cell models, are difficult to analyze by rheometry as the biofilms that form tend to be microscopic and attached to surface interfaces. Together these stretch the limitations of the mechanical instrument and often require disrupting the biofilm in order to transfer it for analysis ^1^. Indeed, we have previously demonstrated that scraping a biofilm from the interface on which it was grown, and combining biomass from multiple biofilms, alters the mechanical properties measurement, compared to those measured from non-disrupted *in situ* biofilms ^2^. It is recognized that colony morphology reflects the biofilm forming capability of bacteria, offering a high-throughput method for the analysis of biofilm surrogate phenotypes ^3-5^. Importantly, colony-biofilms share phenotypes of biofilms grown in more traditional models, including antibiotic tolerance ^6,7^. Therefore, the colony-biofilm model is emerging as an ideal model for the rheological analysis of biofilms ^2,8,9^, as it (i) permits the growth of biofilms with high biomass, required for these analyses; (ii) allows the mechanical analysis of non-disrupted biofilms; and (iii) is a high-throughput method for growing biofilms that share properties to conventional biofilm models. For these reasons, we used the colony-biofilm model here.

**Rheology theory**

To analyze the mechanical properties of *M. abscessus* colony-biofilms we used both uniaxial mechanical indentation and spinning-disc shear rheology. From these analyses, moduli of the biofilm were determined, which are a measurement of the stress-strain relationship exerted by a material that defines the mechanical properties intrinsic to that material ^10^.

During uniaxial indentation, a normal force (force applied perpendicular to a surface) is applied to the biofilm. This compresses the biofilm in the z-plane for a specified distance and the force required to compress the biofilm is recorded. From the resulting force-displacement relationship the Young’s modulus can be determined ^11^. The Young’s modulus describes the extent that a material will resist being compressed by a normal force, and is an indication of how elastic or stiff a material is under this normal force ^11^.

During spinning-disc shear rheology, a shear force (force that is applied parallel to a surface) is applied to the biofilm at either constant or varying forces. The stresses or strains that build up in the biofilm, in response to this shear force, is measured. For viscoelastic materials this response can be separated into two moduli, the storage (G’) and loss (G”) moduli. The storage modulus describes the energy that is stored by a material, or the elastic response, whereas the loss modulus describes the energy that is lost, or the viscous response ^12^. These moduli are defined by the following equations ^12^:

$$G^{'}= \frac{\sigma_{0}}{\gamma_{0}} cos\delta(S1)$$

$$G" = \frac{\sigma_{0}}{\gamma_{0}} sin\delta(S2)$$

where σ_o_ is the stress amplitude, γ_o_ is the strain amplitude and δ is the phase angle.

From spinning-disc analyses, the modulus that is higher for a given stress, strain or frequency, is the dominant response for that material. Relationships between the storage and loss moduli are also useful parameters for understanding viscoelastic behavior. The ratio of the loss modulus to the storage modulus (or the tangent to the phase angle; tanδ) describes the damping, or energy dissipated, by a material. For viscoelastic materials tanδ <1 indicates elastic solid-like behavior, while tanδ >1 indicates viscous fluid-like behavior ^12^. The complex modulus (G*) describes the extent that a material can resist deformation, and is an indication of the rigidity of a material, when exposed to forces within the linear viscoelastic region ^12^. Tanδ and G* are described by Supplementary equations (S3) and (S4) respectively ^12^, and are used in equations 2 and 3 to determine the theoretical mucociliary and cough clearance indices:

$$tan\delta= \frac{G"}{G'} (S3)$$

$$G^{*}=G^{'}+ iG" (S4)$$

where *i* is $\sqrt{-1}$.

**Supplemental Results**

**Rheology testing parameters can influence the measured mechanical behavior of bacterial biofilms**

Here, we also wanted to compare the mechanical properties of *M. abscessus* biofilms to that of another common pulmonary pathogen. We have previously performed an extensive analysis on the mechanical properties of *Pseudomonas aeruginosa* biofilms ^2^, which is also an important pathogen that causes pulmonary infections in people with cystic fibrosis ^13^. We therefore analyzed 4 day wild type *P. aeruginosa* colony-biofilms using the same rheological parameters used to analyze the *M. abscessus* biofilms. Interestingly, under these conditions (37°C and constant strain of 0.1%), *P. aeruginosa* biofilms displayed increased elastic behavior, compared to what we had previously measured (25°C and constant stress of 0.5Pa [approximately 2% strain] ^2^). As such the mucociliary (MCI) and cough (CCI) clearance index for *P. aeruginosa* colony-biofilms determined here, were lower than what we had previously reported ^2^. We therefore repeated the frequency sweep analysis, using the testing parameters of our previous study. Under these conditions the viscoelastic properties and MCI and CCI of *P. aeruginosa* colony-biofilms were similar to those we had previously observed ^2^ (Fig S1B, C), further illustrating how the dynamic nature of biofilm mechanics and how the testing conditions can influence the measured mechanical behaviour.

**Supplemental Figure Legend**

**
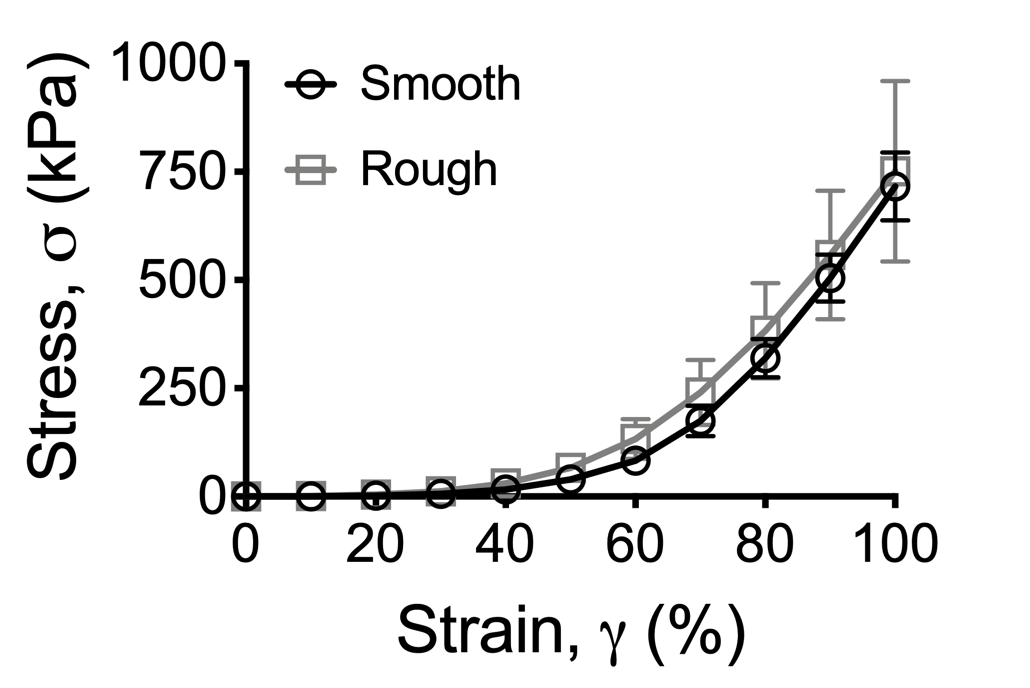
**

**Supplemental Figure 1: Uniaxial mechanical indentation of *M. abscessus* colony-biofilms.** Stress-strain curves of 4 day *M. abscessus* colony-biofilms determined from uniaxial indentation analysis. N=4; data presented as individual data points with mean ± SD.


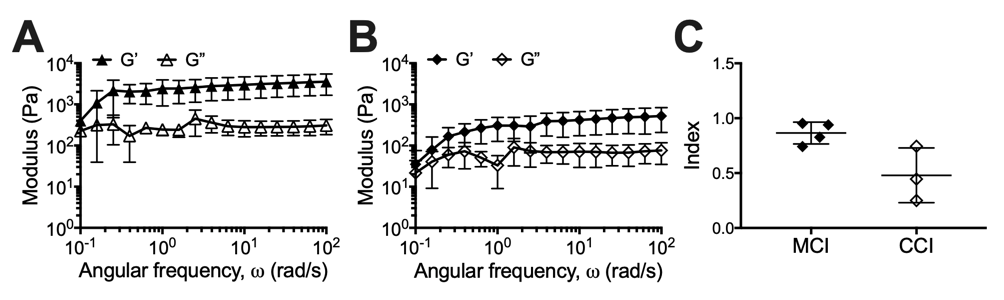


**Supplemental Figure 2: Analysis of *P. aeruginosa* colony-biofilms.** Frequency profiles of wild type *P. aeruginosa* colony-biofilms measured at **(A)** 37°C and strain of 0.1%, as per the analysis of *M. abscessus* biofilms and at **(B)** 25°C and stress of 0.5Pa (equivalent to 1.8 ± 0.4% strain) as per our previous analysis ^2^. **(C)** Mucociliary and cough clearance index of P. aeruginosa colony-biofilms determined from the frequency sweep data in **(B)**.

**Supplemental References**

1 Gloag, E. S., Fabbri, S., Wozniak, D. J. & Stoodley, P. Biofilm mechanics: implications in infection and survival. *Biofilm*, 100017, doi:doi.org/10.1016/j.bioflm.2019.100017 (2019).

2 Gloag, E. S., German, G. K., Stoodley, P. & Wozniak, D. J. Viscoelastic properties of Pseudomonas aeruginosa variant biofilms. *Scientific reports* **8**, 9691, doi:10.1038/s41598-018-28009-5 (2018).

3 Branda, S. S., Vik, A., Friedman, L. & Kolter, R. Biofilms: the matrix revisited. *Trends Microbiol.* **13**, 20-26 (2005).

4 Haussler, S. & Fuqua, C. Biofilms 2012: new discoveries and significant wrinkles in a dynamic field. *J Bacteriol* **195**, 2947-2958, doi:10.1128/jb.00239-13 (2013).

5 Evans, T. J. Small colony variants of Pseudomonas aeruginosa in chronic bacterial infection of the lung in cystic fibrosis. *Future microbiology* **10**, 231-239, doi:10.2217/fmb.14.107 (2015).

6 Anderl, J. N., Franklin, M. J. & Stewart, P. S. Role of antibiotic penetration limitation in *Klebsiella pneumoniae* biofilm resistance to ampicillin and ciprofloxacin. *Antimicrobial agents and chemotherapy* **44**, 1818-1824 (2000).

7 Walters, M. C., Roe, F., Bugnicourt, A., Franklin, M. J. & Stewart, P. S. Contributions of antibiotic penetration, oxygen limitation, and low metabolic activity to tolerance of Pseudomonas aeruginosa biofilms to ciprofloxacin and tobramycin. *Antimicrobial agents and chemotherapy* **47**, 317-323 (2003).

8 Jones, W. L., Sutton, M. P., McKittrick, L. & Stewart, P. S. Chemical and antimicrobial treatments change the viscoelastic properties of bacterial biofilms. *Biofouling* **27**, 207-215, doi:10.1080/08927014.2011.554977 (2011).

9 Wloka, M., Rehage, H., Flemming, H.-C. & Wingender, J. Structure and rheological behaviour of the extracellular polymeric substance network of mucoid Pseudomonas aeruginosa biofilms. *Biofilms* **2**, 275-283 (2005).

10 Vincent, J. F. *Structural biomaterials*. (Princeton University Press, 2012).

11 Timoshenko, S. & Goodier, J. *Theory of Elasticity*. third edn, (McGraw Hill Higher Education, 1970).

12 Ferry, J. D. *Viscoelastic properties of polymers*. (John Wiley & Sons, 1980).

13 Ciofu, O., Tolker-Nielsen, T., Jensen, P. O., Wang, H. & Hoiby, N. Antimicrobial resistance, respiratory tract infections and role of biofilms in lung infections in cystic fibrosis patients. *Advanced drug delivery reviews* **85**, 7-23, doi:10.1016/j.addr.2014.11.017 (2015).
